# Poly[bis­(μ_2_-1,3-phenyl­enedi­amine-κ^2^ *N*:*N*′)di-μ-thio­cyanato-κ^2^ *N*:*S*;κ^2^ *S*:*N*-cadmium]

**DOI:** 10.1107/S1600536813031255

**Published:** 2013-11-20

**Authors:** Rakia Chemli, Slaheddine Kamoun, Thierry Roisnel

**Affiliations:** a Laboratoire de Génie des Matériaux et Environnement, École Nationale d’Ingénieurs de Sfax, BP 1173, Sfax, Tunisia; b Centre de Diffractométrie X, UMR 6226 CNRS Unité des Sciences Chimiques de Rennes, Université de Rennes I, 263 Avenue du Général Leclerc, 35042 Rennes, France

## Abstract

The structure of the title polymeric compound, [Cd(SCN)_2_(C_6_H_8_N_2_)_2_]_*n*_, exhibits a two-dimensional staircase-like structure parallel to (010) in which the Cd^II^ atom lies on a twofold rotation axis and has a distorted octa­hedral CdS_2_N_4_ geometry involving four μ-1,3-(SCN) group donors and two N-atom donors from 1,3-phenyl­enedi­amine ligands, which also have twofold symmetry. The major contributions to the cohesion and the stability of this two-dimensional polymeric structure are the covalent Cd—S,N bonds and one weak intra­layer N—H⋯S hydrogen bond.

## Related literature

For related structures, see: MacGillivary *et al.* (1994 ▶); Fujita *et al.* (1995 ▶); Blake *et al.* (1997 ▶); Withersby *et al.* (1997 ▶); Tong *et al.* (1998 ▶); Yang *et al.* (2001 ▶); Chemli *et al.* (2013 ▶). For the HSCN synthesis, see: Bartlett *et al.* (1969 ▶). For the effects of substituents on the inter­nal angles of the phenyl ring, see: Domenicano & Murray-Rust (1979 ▶). For NLO and luminescence of related compounds, see: Chen *et al.* (2000 ▶); Bai *et al.* (2011 ▶). For electric and dielectric properties of related compounds, see: Karoui *et al.* (2013 ▶).
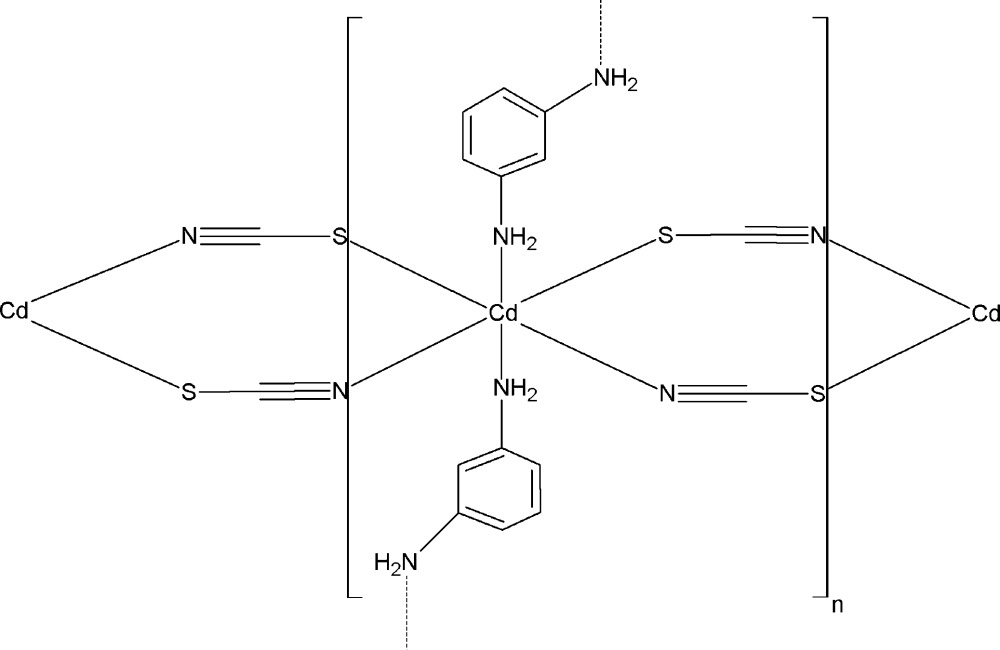


## Experimental

### 

#### Crystal data

[Cd(NCS)_2_(C_6_H_8_N_2_)_2_]
*M* *_r_* = 336.7
Monoclinic, 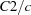

*a* = 10.8704 (6) Å
*b* = 12.8983 (10) Å
*c* = 8.3362 (5) Å
β = 106.503 (3)°
*V* = 1120.67 (13) Å^3^
*Z* = 4
Mo *K*α radiation
μ = 2.29 mm^−1^
*T* = 150 K
0.17 × 0.07 × 0.06 mm

#### Data collection

Bruker APEXII diffractometer
Absorption correction: multi-scan (*SADABS*; Bruker, 2011 ▶) *T*_min_ = 0.825, *T*_max_ = 0.872
4396 measured reflections
1283 independent reflections
1130 reflections with *I* > 2σ(*I*)
*R*_int_ = 0.061

#### Refinement

*R*[*F* ^2^ > 2σ(*F* ^2^)] = 0.034
*wR*(*F* ^2^) = 0.064
*S* = 1.03
1283 reflections
70 parameters
H-atom parameters constrained
Δρ_max_ = 0.60 e Å^−3^
Δρ_min_ = −0.58 e Å^−3^

### 

Data collection: *APEX2* (Bruker, 2011 ▶); cell refinement: *SAINT* (Bruker, 2011 ▶); data reduction: *SAINT*; program(s) used to solve structure: *SHELXS97* (Sheldrick, 2008 ▶); program(s) used to refine structure: *SHELXL97* (Sheldrick, 2008 ▶); molecular graphics: *DIAMOND* (Brandenburg & Berndt, 2001 ▶) and *Mercury* (Macrae *et al.*, 2008 ▶); software used to prepare material for publication: *WinGX* (Farrugia, 2012) ▶ and *publCIF* (Westrip, 2010 ▶).

## Supplementary Material

Crystal structure: contains datablock(s) I. DOI: 10.1107/S1600536813031255/vn2078sup1.cif

Structure factors: contains datablock(s) I. DOI: 10.1107/S1600536813031255/vn2078Isup2.hkl

Supplementary material file. DOI: 10.1107/S1600536813031255/vn2078Isup3.cdx

Additional supplementary materials:  crystallographic information; 3D view; checkCIF report

## Figures and Tables

**Table 1:** Selected bond lengths (Å)

Cd1—N1^i^	2.306 (3)
Cd1—N2	2.364 (3)
Cd1—S1	2.7143 (10)

Symmetry code: (i) 
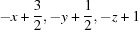
.

**Table 2:** Hydrogen-bond geometry (Å, °)

*D*—H⋯*A*	*D*—H	H⋯*A*	*D*⋯*A*	*D*—H⋯*A*
N2—H2*B*⋯S1^iii^	0.92	2.67	3.589 (3)	173

Symmetry code: (iii) 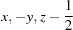.

**Fig. 1.**
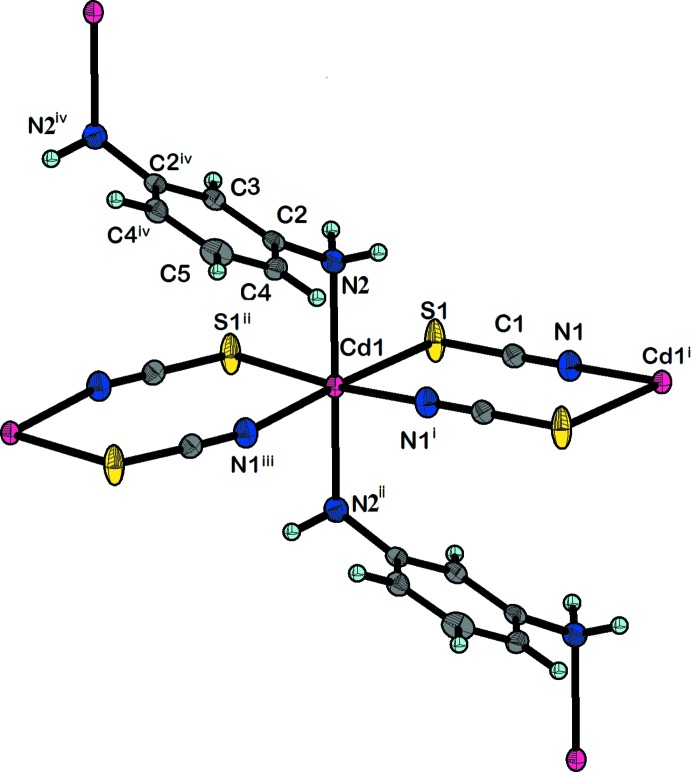
The molecular structure of the title compound, showing the coordination around the Cd2+ cations with labelling and displacement ellipsoids drawn at the 50% probability level. The H atoms are omitted for clarity. Symmetry codes: (i):-x + 3/2, -y + 1/2, -z + 1; (ii):x - 1/2, -y + 1/2, z - 1/2;(iii):-x + 1, y, -z + 1/2; (iv):-x + 1, y, -z - 1/2

**Fig. 2.**
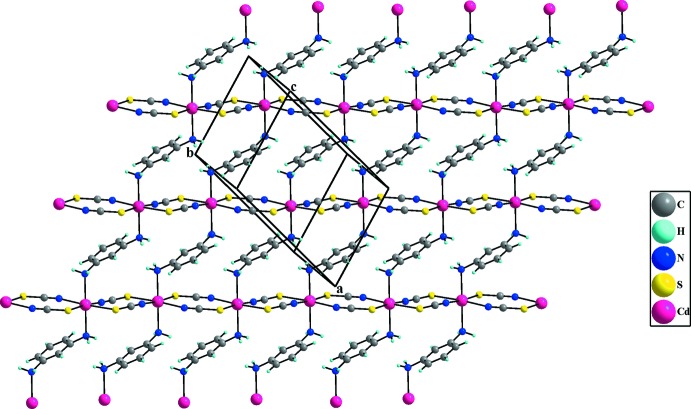
The two-dimensional molecular structure of the title coordination polymer showing the staircase molecular arrangement.

## supplementary crystallographic information

### 1. Comment

The crystal engineering of inorganic-organic hybrid coordination polymers is currently one of the most active fields in coordination chemistry, supramolecular and materials chemistry. These compounds attract significant attention for their architectures and topologies (Yang *et al.*, 2001), including a large number of extended assemblies such as helical network (Withersby *et al.*, 1997; Chemli *et al.* 2013) molecular zippers, diamondoid, honeycomb (Tong *et al.*, 1998), square- grid (MacGillivary *et al.*, 1994), T-shaped and ladder frameworks. (Fujita *et al.*, 1995; Blake *et al.*,1997). Hybrid inorganic-organic thiocyanate materials exhibit interesting physical properties such electrical conductivity and dielectric relaxation process (Karoui *et al.*, 2013) and may have potential applications in non-linear optics and luminescence (Chen *et al.*, 2000; Bai *et al.*, 2011). Herein we report the structure of a new polymeric hybrid [Cd(SCN)_2_(C_6_H_8_N_2_)_2_]_n_. As shown in Fig. 1, each cadmium atom, which sits on a twofold rotation axis, is coordinated by two *cis* N-bonded and two *cis* S-bonded thiocyanato anions. Two *trans*-coordinated neutral *m*-phenylenediamine ligands complete the octahedral coordination geometry around cadmium. The crystal structure of the title compound consists of both doubly µ-1,3-SCN and µ-1,3-phenylendiamine –bridged two-dimensional networks (Fig. 2). In the CdN4S2 core, the Cd—N and Cd—S bonds are in the range 2.3061 - 2.364 (3) Å and 2.7143 (10) Å, respectively (Table 1). The bond angles involving the cadmium (II) atom range from 83.89 (11) to 96.91 (10)° and from 174.75 (8) to 178.87 (14)°. These values are in good agreement with those observed in other similar complexes (Chemli *et al.* 2013). The double SCN bridging mode gives rise to a centrosymmetrical eight-membered Cd(SCN)_2_Cd rings in a chair conformation because of the almost linear SCN groups (S–C–N angle = 178.8 (3)°). The distance between adjacent Cd atoms in Cd_2_ (SCN)_2_ rings is 5.937 Å. Again, these rings built up a staircase-like chain through their corner-sharing action at the two cadmium atoms *via* the µ-1,3-phenylenediamine moiety and give rise to twenty-membered [Cd_4_(µ-1,3-SCN)_4_(µ-1,3-phenylendiamine)_2_] macrocycles as subunits, as depicted in Fig. 2. The bond angles in the phenyl groups deviate significantly from the idealized value of 120° due to the effect of the substituent. In fact, it was established that the angular deformations of phenyl groups can be described as a sum of the effects of the different substituents (Domenicano & Murray-Rust, 1979). The phenyl rings of 1,3-phenylendiamine ligand are planar with the greatest deviation from the six-atoms least-square plane of 0.0001 Å. They are well ordered with C–C–C angles in agreement with the expected *sp*^2^ hybridization. The π-π interactions between neighboring phenyl rings may be neglected (>4 Å). The major contributions of the cohesion and the stability of this polymeric structure is assured by the covalent Cd-(S,*N*) bonds and the presence of one weak intralayer N–H···S hydrogen bond with the H···S and N···S distances of 2.674 Å and 3.589 (3) Å, respectively (Table 2).

### 2. Experimental

To 25 ml of an aqueous solution of thiocyanic acid (0.4 mol.l^-1^) prepared using the published procedure (Bartlett *et al.*, 1969) an appropriate amount of cadmium carbonate (0.832 g, 5 mmol) was added and refluxed for 2 h. After cooling, 25 ml of methanol and 0.7 ml of a solution of 1,3-phenylenediamine (7.5 mol.l^-1^) was added. The resulting solution was heated under reflux for 2 h and left at ambient temperature for 2 hours after which well shaped brown crystals were obtained on slow evaporation of the solvent. They were washed with diethyl ether and dried over P_2_O_5_.

### 3. Refinement

All H atoms were positioned geometrically and allowed to ride on their parent atoms, with C—H = 0.95–0.98 Å, N—H = 0.92 Å, and with *U*_iso_(H) = 1.2*U*_eq_(C, N).

### Crystal data

**Table:**

[Cd(NCS)_2_(C_6_H_8_N_2_)_2_]	*F*(000) = 656
*M**_r_* = 336.7	*D*_x_ = 1.996 Mg m^−^^3^*D*_m_ = 1.931 Mg m^−^^3^*D*_m_ measured by Flotation
Monoclinic, *C*2/*c*	Mo *K*α radiation, λ = 0.71073 Å
Hall symbol: -C 2yc	Cell parameters from 1283 reflections
*a* = 10.8704 (6) Å	θ = 3.2–27.5°
*b* = 12.8983 (10) Å	µ = 2.29 mm^−^^1^
*c* = 8.3362 (5) Å	*T* = 150 K
β = 106.503 (3)°	Prism, brown
*V* = 1120.67 (13) Å^3^	0.17 × 0.07 × 0.06 mm
*Z* = 4	

### Data collection

**Table:**

Bruker APEXII diffractometer	1130 reflections with *I* > 2σ(*I*)
Radiation source: fine-focus sealed tube	*R*_int_ = 0.061
Graphite monochromator	θ_max_ = 27.5°, θ_min_ = 3.2°
CCD rotation images, thin slices scans	*h* = −10→14
Absorption correction: multi-scan (*SADABS*; Bruker, 2011)	*k* = −16→16
*T*_min_ = 0.825, *T*_max_ = 0.872	*l* = −10→9
4396 measured reflections	2 standard reflections every 120 min
1283 independent reflections	

### Refinement

**Table:**

Refinement on *F*^2^	Primary atom site location: structure-invariant direct methods
Least-squares matrix: full	Secondary atom site location: difference Fourier map
*R*[*F*^2^ > 2σ(*F*^2^)] = 0.034	Hydrogen site location: inferred from neighbouring sites
*wR*(*F*^2^) = 0.064	H-atom parameters constrained
*S* = 1.03	*w* = 1/[σ^2^(*F*_o_^2^) + (0.0156*P*)^2^] where *P* = (*F*_o_^2^ + 2*F*_c_^2^)/3
1283 reflections	(Δ/σ)_max_ < 0.001
70 parameters	Δρ_max_ = 0.60 e Å^−^^3^
0 restraints	Δρ_min_ = −0.58 e Å^−^^3^

### Special details

**Table:**

Geometry. All e.s.d.'s (except the e.s.d. in the dihedral angle between two l.s. planes) are estimated using the full covariance matrix. The cell e.s.d.'s are taken into account individually in the estimation of e.s.d.'s in distances, angles and torsion angles; correlations between e.s.d.'s in cell parameters are only used when they are defined by crystal symmetry. An approximate (isotropic) treatment of cell e.s.d.'s is used for estimating e.s.d.'s involving l.s. planes.
Refinement. Refinement of *F*^2^ against ALL reflections. The weighted *R*-factor *wR* and goodness of fit *S* are based on *F*^2^, conventional *R*-factors *R* are based on *F*, with *F* set to zero for negative *F*^2^. The threshold expression of *F*^2^ > σ(*F*^2^) is used only for calculating *R*-factors(gt) *etc*. and is not relevant to the choice of reflections for refinement. *R*-factors based on *F*^2^ are statistically about twice as large as those based on *F*, and *R*- factors based on ALL data will be even larger.

### Fractional atomic coordinates and isotropic or equivalent isotropic displacement parameters (Å^2^)

**Table:**

	*x*	*y*	*z*	*U*_iso_*/*U*_eq_	
Cd1	0.5	0.20749 (3)	0.25	0.01462 (12)	
S1	0.65559 (10)	0.05195 (8)	0.40889 (13)	0.0297 (3)	
N1	0.8532 (3)	0.1670 (2)	0.6240 (4)	0.0219 (7)	
C1	0.7707 (3)	0.1202 (3)	0.5352 (4)	0.0163 (8)	
N2	0.6151 (3)	0.2057 (2)	0.0483 (4)	0.0191 (7)	
H2A	0.6952	0.233	0.0968	0.023*	
H2B	0.6263	0.1377	0.0223	0.023*	
C2	0.5594 (3)	0.2606 (3)	−0.1055 (4)	0.0159 (8)	
C3	0.5	0.2075 (4)	−0.25	0.0149 (10)	
H3	0.5	0.1339	−0.25	0.018*	
C4	0.5598 (3)	0.3684 (3)	−0.1044 (4)	0.0174 (8)	
H4	0.6007	0.4054	−0.0049	0.021*	
C5	0.5	0.4211 (4)	−0.25	0.0237 (13)	
H5	0.5	0.4947	−0.25	0.028*	

### Atomic displacement parameters (Å^2^)

**Table:**

	*U*^11^	*U*^22^	*U*^33^	*U*^12^	*U*^13^	*U*^23^
Cd1	0.0134 (2)	0.0146 (2)	0.0142 (2)	0	0.00127 (14)	0
S1	0.0252 (6)	0.0127 (5)	0.0371 (6)	−0.0027 (4)	−0.0140 (4)	0.0019 (4)
N1	0.0209 (18)	0.0165 (17)	0.0248 (18)	0.0001 (14)	0.0010 (15)	0.0012 (14)
C1	0.0176 (19)	0.0127 (19)	0.0180 (19)	0.0044 (16)	0.0041 (15)	0.0024 (15)
N2	0.0182 (16)	0.0191 (17)	0.0199 (17)	0.0006 (14)	0.0051 (13)	−0.0011 (14)
C2	0.0117 (18)	0.021 (2)	0.0168 (19)	0.0008 (15)	0.0067 (15)	0.0013 (15)
C3	0.013 (2)	0.012 (3)	0.020 (3)	0	0.006 (2)	0
C4	0.0166 (19)	0.018 (2)	0.0175 (19)	−0.0036 (15)	0.0046 (15)	−0.0055 (15)
C5	0.028 (3)	0.010 (3)	0.036 (3)	0	0.014 (3)	0

### Geometric parameters (Å, º)

**Table:**

Cd1—N1^i^	2.306 (3)	N2—H2A	0.92
Cd1—N1^ii^	2.306 (3)	N2—H2B	0.92
Cd1—N2^iii^	2.364 (3)	C2—C3	1.376 (4)
Cd1—N2	2.364 (3)	C2—C4	1.391 (5)
Cd1—S1^iii^	2.7143 (10)	C3—C2^iv^	1.376 (4)
Cd1—S1	2.7143 (10)	C3—H3	0.95
S1—C1	1.643 (4)	C4—C5	1.382 (4)
N1—C1	1.157 (4)	C4—H4	0.95
N1—Cd1^i^	2.306 (3)	C5—C4^iv^	1.382 (4)
N2—C2	1.439 (4)	C5—H5	0.95
			
N1^i^—Cd1—N1^ii^	90.81 (15)	C2—N2—Cd1	117.1 (2)
N1^i^—Cd1—N2^iii^	96.91 (10)	C2—N2—H2A	108
N1^ii^—Cd1—N2^iii^	83.89 (11)	Cd1—N2—H2A	108
N1^i^—Cd1—N2	83.89 (11)	C2—N2—H2B	108
N1^ii^—Cd1—N2	96.91 (10)	Cd1—N2—H2B	108
N2^iii^—Cd1—N2	178.87 (14)	H2A—N2—H2B	107.3
N1^i^—Cd1—S1^iii^	174.75 (8)	C3—C2—C4	120.2 (4)
N1^ii^—Cd1—S1^iii^	92.41 (8)	C3—C2—N2	120.6 (3)
N2^iii^—Cd1—S1^iii^	87.56 (8)	C4—C2—N2	119.1 (3)
N2—Cd1—S1^iii^	91.60 (8)	C2—C3—C2^iv^	120.4 (5)
N1^i^—Cd1—S1	92.41 (8)	C2—C3—H3	119.8
N1^ii^—Cd1—S1	174.75 (8)	C2^iv^—C3—H3	119.8
N2^iii^—Cd1—S1	91.60 (8)	C5—C4—C2	119.0 (4)
N2—Cd1—S1	87.56 (8)	C5—C4—H4	120.5
S1^iii^—Cd1—S1	84.68 (4)	C2—C4—H4	120.5
C1—S1—Cd1	99.92 (12)	C4^iv^—C5—C4	121.2 (5)
C1—N1—Cd1^i^	165.4 (3)	C4^iv^—C5—H5	119.4
N1—C1—S1	178.8 (3)	C4—C5—H5	119.4
			
N1^i^—Cd1—S1—C1	6.69 (14)	S1^iii^—Cd1—N2—C2	81.6 (2)
N1^ii^—Cd1—S1—C1	−121.1 (9)	S1—Cd1—N2—C2	166.2 (2)
N2^iii^—Cd1—S1—C1	−90.29 (14)	Cd1—N2—C2—C3	−103.9 (3)
N2—Cd1—S1—C1	90.47 (15)	Cd1—N2—C2—C4	72.5 (3)
S1^iii^—Cd1—S1—C1	−177.70 (14)	C4—C2—C3—C2^iv^	0.0 (2)
Cd1^i^—N1—C1—S1	122 (17)	N2—C2—C3—C2^iv^	176.3 (3)
Cd1—S1—C1—N1	−147 (17)	C3—C2—C4—C5	0.0 (4)
N1^i^—Cd1—N2—C2	−101.1 (2)	N2—C2—C4—C5	−176.4 (3)
N1^ii^—Cd1—N2—C2	−11.0 (3)	C2—C4—C5—C4^iv^	0.0 (2)
N2^iii^—Cd1—N2—C2	123.9 (2)		

Symmetry codes: (i) −*x*+3/2, −*y*+1/2, −*z*+1; (ii) *x*−1/2, −*y*+1/2, *z*−1/2; (iii) −*x*+1, *y*, −*z*+1/2; (iv) −*x*+1, *y*, −*z*−1/2.

### Hydrogen-bond geometry (Å, º)

**Table:**

*D*—H···*A*	*D*—H	H···*A*	*D*···*A*	*D*—H···*A*
N2—H2*B*···S1^v^	0.92	2.67	3.589 (3)	173

Symmetry code: (v) *x*, −*y*, *z*−1/2.

## References

[bb1] Bai, Y., Hu, X., Dang, D., Bi, F. & Niu, J. (2011). *Spectrochim. Acta*, **78**, 70–73. 10.1016/j.saa.2010.08.066 20934904

[bb2] Bartlett, H. E., Jurriaanse, A. & De Haas, K. (1969). *Can. J. Chem.* **47**, 16, 2981–2986.

[bb3] Blake, A. J., Champness, N. R., Khlobystov, A., Lemenovskii, D. A., Li, W.-S. & Schroder, M. (1997). *Chem. Commun.* pp. 2027–2028.

[bb4] Brandenburg, K. & Berndt, M. (2001). *DIAMOND* Crystal Impact, Bonn, Germany.

[bb5] Bruker (2011). *APEX2*, *SAINT* and *SADABS* Bruker AXS Inc., Madison, Wisconsin, USA.

[bb6] Chemli, R., Kamoun, S. & Roisnel, T. (2013). *Acta Cryst.* E**69**, m292–m293. 10.1107/S1600536813010738 PMC3647823 23723789

[bb7] Chen, H., Zhang, L., Cai, Z., Guang Yanga, G. & Chen, X. (2000). *J. Chem. Soc. Dalton Trans.* pp. 2463–2466.

[bb8] Domenicano, A. & Murray-Rust, P. (1979). *Tetrahedron Lett.* **24**, 2283–2286.

[bb9] Farrugia, L. J. (2012). *J. Appl. Cryst.* **45**, 849–854.

[bb10] Fujita, M., Kwan, Y. J., Sataki, O., Yamaguchi, K. & Ogura, K. (1995). *J. Am. Chem. Soc.* **117**, 7287–7288.

[bb11] Karoui, S., Kamoun, S. & Jouini, A. (2013). *J. Solid State Chem.* **197**, 60–68.

[bb12] MacGillivary, L. R., Subramamian, S. & Zaworotko, M. J. (1994). *J. Chem. Soc. Chem. Commun.* pp. 1325–1326.

[bb13] Macrae, C. F., Bruno, I. J., Chisholm, J. A., Edgington, P. R., McCabe, P., Pidcock, E., Rodriguez-Monge, L., Taylor, R., van de Streek, J. & Wood, P. A. (2008). *J. Appl. Cryst.* **41**, 466–470.

[bb14] Sheldrick, G. M. (2008). *Acta Cryst.* A**64**, 112–122. 10.1107/S0108767307043930 18156677

[bb15] Tong, M.-L., Ye, B.-H., Cai, J.-W. & Chen, X.-M. (1998). *Inorg. Chem.* **37**, 2645–2650. 10.1021/ic9714293 11670398

[bb16] Westrip, S. P. (2010). *J. Appl. Cryst.* **43**, 920–925.

[bb17] Withersby, M. A., Blake, A. J., Champness, N. R., Hubberstey, P., Li, W.-S. & Schroder, M. (1997). *Angew. Chem. Int. Ed. Engl.* **36**, 2327–2329.

[bb18] Yang, G., Zhu, H.-G., Liang, B.-H. & Chen, X.-M. (2001). *J. Chem. Soc* *Dalton Trans* pp. 580–585.

